# Efficacy of the decolonization of methicillin-resistant *Staphylococcus aureus* carriers in clinical practice

**DOI:** 10.1186/s13756-015-0096-x

**Published:** 2015-12-18

**Authors:** N. Sai, C. Laurent, H. Strale, O. Denis, B. Byl

**Affiliations:** Hospital Epidemiology and Infection Control Unit, Erasme Hospital, Université Libre de Bruxelles, 808, Route de Lennik, 1070 Brussels, Belgium; Hygiène Hospitalière, Centre Hospitalier Régional de Namur, Namur, Belgium; Department of Clinical Microbiology, Erasme Hospital, Université Libre de Bruxelles, Brussels, Belgium; School of Public Health, Université Libre de Bruxelles, Brussels, Belgium

## Abstract

**Background:**

Nasal and extra nasal carriage of methicillin-resistant *S. aureus* (MRSA) is a pre-existing condition that often leads to invasive MRSA infection, as MRSA colonization is associated with a high risk of acquiring MRSA infection during hospital stays. Decolonization may reduce the risk of meticillin-resistant Staphylococcus aureus (MRSA) infection in individual carriers and prevent transmission to other patients.

**Methods:**

A retrospective cohort study was conducted to evaluate the effectiveness of two decolonization protocols for newly diagnosed MRSA carriage in hospitalized patients and to assess the impact of decolonization on the rate of MRSA infection. The study population consisted of all patients diagnosed as MRSA-positive between January 2006 and June 2010.

Patients diagnosed as carriers were designated as requiring contact precautions by the hospital infection control team. The standing order protocol of the hospital pertaining to decolonization procedures was then applied, and all newly diagnosed patients were administered one of the two decolonization treatments outlined in the hospital protocol, with the exception of MRSA respiratory carriers (MRSA obtained from sputum or other lower respiratory tract samples). The two decolonization treatments consisted of the application of intranasal mupirocin 2 % and washing with chlorhexidine soap (40 mg/mL) (mupi/CHX) or application of intranasal povidone-iodine and washing with povidone-iodine soap (PVPI), with each treatment lasting for 5 days.

Success was determined by at least three successive nose swabs and throat and other screened site swabs that tested negative for MRSA before patient discharge.

**Results:**

A total of 1150 patients admitted to the hospital were found to be infected or colonized with MRSA. Of the 1150 patients, 268 were prescribed decolonization treatment. 104 out of 268 patients (39 %) were successfully decolonized. There was no significant success after two decolonization failures.

MRSA infection rate among the successes and failures were 0.0 and 4.3 %, respectively [*P* = 0.04].

**Conclusions:**

Our results fit well with the prescription of decolonization based on local strategy protocols but reflect a low rate of successful treatment.

Although the success rate of decolonization was not high in our study, the effectiveness of decolonization on the infection rate, justifies the continuation of this strategy, even if a marginal cost is incurred.

## Background

The burden of methicillin-resistant *Staphylococcus aureus* (MRSA) infection remains high, with approximately 100 000 invasive MRSA infections occurring annually in the United States causing approximately 19 000 deaths — more than that caused by HIV, for example [1]. Moreover, treatment options for MRSA are limited, most often require intravenous access, have greater side effects and are more expensive than standard treatments [2].

Nasal and extra nasal carriage of methicillin-resistant *S. aureus* (MRSA) often leads to invasive MRSA infection, as MRSA colonization is associated with a risk of up to 30 % of acquiring MRSA infection during hospital stays [3, 4]. Moreover, carriers of MRSA serve as reservoirs in hospitals and long-term care facilities, and the pressure of colonization plays an important role in the subsequent dissemination of MRSA strains in these institutions [5].

In contrast to other resistant or difficult to treat microorganisms, MRSA carriage can be eliminated through the application of decolonization agents, such as nasal mupirocin and chlorexhidine soap. Several prospective studies have shown that the optimization of carriage eradication can be effective in controlling MRSA dissemination [6, 7]. A randomized controlled double blind trial showed that The MRSA eradication was 25 % in the mupirocin group in comparison with placebo. The results suggest that nasal mupirocin is only marginally effective in the eradication of multisite MRSA carriage in a setting where MRSA is endemic [8].

It appers from a metanalysis that there is insufficient evidence to support use of topical or systemic antimicrobial nasal or extra-nasal MRSA. There is no demonstrated superiority of either or systemic therapy, or a combination of these agents [9].

Little is known, however, about the rate of success of decolonization strategies under real-life conditions. Moreover, many unresolved issues remain, including the number of unsuccessful eradication attempts that should be administered before concluding failure or the collateral benefit of such decolonization procedures, even if unsuccessful, on reducing infection rates.

## Objective

The aim of this study is to evaluate the effectiveness of two decolonization protocols for newly diagnosed MRSA colonization in hospitalized patients and to assess the impact of decolonization on the rate of MRSA infection.

## Methods

### Setting

Erasme Hospital is the 864-bed academic hospital of the Université Libre de Bruxelles (Brussels, Belgium). The hospital admits, on average, 30,000 patients annually. An MRSA surveillance and control program was initiated in the hospital in 1990 and continues presently in accordance with national guidelines.

Screening for MRSA via swabbing of nose and throat completed by wounds swabbing is routinely performed for patients with a previous history of MRSA colonization or who harbor one of more of the following conditions: patients who have been previously hospitalized or undergone antibiotic treatment within the past six months, patients admitted from other hospitals or long-term care facilities, and patients with wounds, skin lesions or foreign material. Screening is performed twice weekly in patients in intensive care units. MRSA decolonization is offered for a maximum number of patients for both the control of dissemination and to reduce the rate of infection.

### Study design and definitions

**Fig. 1:**
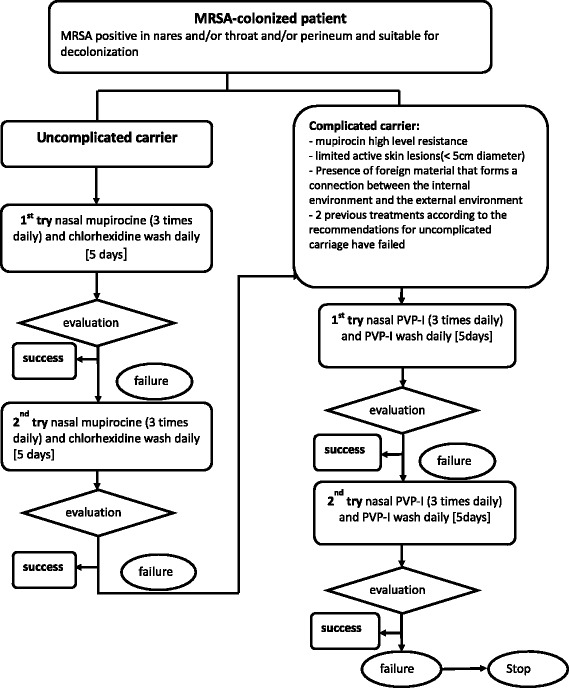
Routine MRSA decolonization protocols employed during the study

A retrospective cohort study was conducted to assess the efficacy of MRSA decolonization and to evaluate the impact of two decolonization protocols on the rate of MRSA infection in patients newly diagnosed as colonized with MRSA. Patients diagnosed as colonized were designated as requiring contact precautions by the hospital infection control team. The standing order protocol of the hospital pertaining to decolonization procedures was then applied, and all newly diagnosed patients were administered a decolonization treatment outlined in the hospital protocol, with the exception of MRSA respiratory carriers (i.e., MRSA obtained from sputum or other lower respiratory tract samples). The two decolonization treatments consisted of the application of intranasal mupirocin 2 % and washing with chlorhexidine soap (40 mg/mL) (mupi/CHX) or application of intranasal povidone-iodine and washing with povidone-iodine soap (PVPI), with each treatment lasting for 5 days. Mupi/CHX was used for uncomplicated cases, whereas PVPI was used for more complicated cases, as defined in Fig. 1. Patients who failed to respond to two mupi/CHX failures were then treated with PVPI. Success was determined by at least three successive nose swabs and throat and other screened site swabs that tested negative for MRSA before the patient could be discharged from the hospital. There was at least 48 h between each swab.

### Study population

The study population consisted of all patients diagnosed as MRSA-positive between January 2006 and June 2010. Information collected included demographic characteristics, transfer(s) from another hospital or long-term care facility, antibiotic use, co-morbidities, site(s) of colonization or infection, date(s) of first colonization, date and decolonization treatment, effectiveness of decolonization procedures, subsequent re-occurrence of MRSA infection, date and site of infection (if any) and date of discharge from the hospital. Patients for whom a decolonization treatment was not prescribed were classified in the no treatment group.

### MRSA identification

Swabs were plated separately onto selective chromogenic MRSA agar (chromID MRSA medium, bioMérieux, Marcy l’Etoile, France) and inoculated into brain–heart infusion broth (BHIB) supplemented with 7.5 % NaCl, and then stored at 4 °C for further analysis. Samples were incubated overnight, and then, the BHIB was subcultured onto chromID MRSA agar. Selective plates were incubated at 35 °C for 48 h and examined daily. Suspected MRSA colonies were identified by the coagulase test using human plasma. Resistance to oxacillin was determined via the cefoxitin disk diffusion method (BD, Oxoid, UK) according to Clinical and Laboratory Standards Institute (CLSI) recommendations. Staphylococcal Cassette Chromosome mec (SCCmec) presence were determined by PCR.

### Statistical analysis

Statistical analysis was performed using Epi Info™ 7 (Centers for Disease Control and Prevention, Atlanta, GA, USA). Normally distributed continuous variables were compared using a 2‐sample t test, while categorical data were compared using a *χ*^2^ test with a Yates correction.

## Results

A total of 1150 patients admitted to the hospital (0.8 % of admissions) were found to be infected or colonized with MRSA over the study period (2006–2010). Of these, MRSA carriage was determined to be imported in 67 % (772) of the patients and hospital-acquired in 33 % (378) of the patients.

Of the 1150 patients, 708 were excluded from the study because of incomplete follow-up for one of more of the following reasons: 335 were excluded before the decolonization treatment was implemented due to patient death (43) or because they were earlier discharged from the hospital (292), while 373 were deemed inevaluable because their hospital stay was too short for the decolonization treatment to be completed and evaluated (328 of the 373), patient death during this period (34 of the 373), or for other reasons (11 of the 373).

Complete follow-up data were available for 442 patients screened by infection control nurses and considered eligible for decolonization treatment (Table 1). However, decolonization treatment was not proposed for 174 (39 %) of these patients because they did not meet one or more of the local-protocol inclusion criteria.

**Table 1:** Characteristics of patients with methicillin-resistant *Staphylococcus aureus* eligible for decolonization between January 2006 and June 2010 (*n* = 442)

	Proportion of patients		
	Patients treated	Patients not treated	*P*
	(*n* = 268)	(*n* = 174)	
Sex	Male 142 (53)	104 (60)	.160
	Female 126 (47)	70 (40)	
Age, mean, years ± SD	69.09 ± 16.52	69 ± 17.26	.9
Length of hospital stay mean, years ± SD	48.54 ± 35.71	36.91 ± 32.99	<.001
Type of MRSA colonization			
Nosocomial	107 (39.9)	88 (50)	.04
Site of colonization			
Nose	147 (55)	53 (26.5)	<.001
Throat	115 (43)	43 (25)	<.001
Perineum	116 (43)	39 (25)	<.001
Others	109 (41)	146 (84)	<.001
Wound	85 (31.7)	46 (26.4)	.2
Number of colonized sites			
Mean, no ± SD	1.8 ± 1	1.6 ± 0.94	.03
≥2sites	128 (47.8)	64 (36.8)	.02
Comorbidity (underlying condition)			
Immunosuppressive therapy	36 (13.4)	39 (52)	.01
Diabetes	71 (65)	38 (35)	.26
Urine catheter	14 (5.2)	6 (3.4)	.48
Dialysis	11 (4.1)	4 (2.3)	.30
CVC	9 (3.3)	10 (5.7)	.22
COPD	31 (11.5)	33 (18.9)	.003

*Note*: data are no (%) of patients

**Fig. 2:**
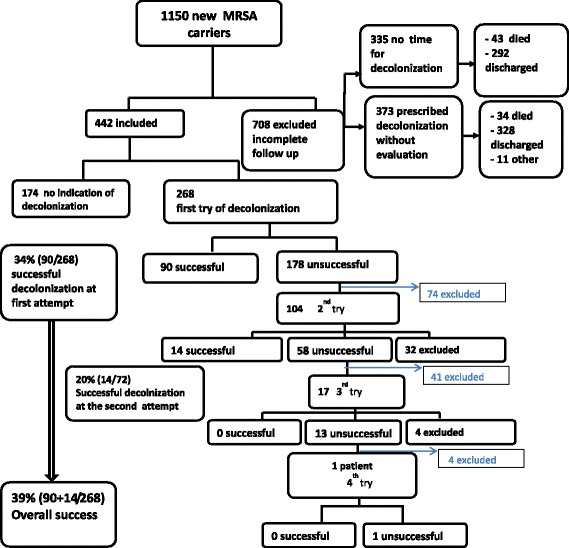
Decolonization results

Of the 268 patients who were prescribed decolonization treatment (Fig. 2), 34 % (90/268) were cleared of MRSA infection at the first attempt and 20 % (14/72) at the second attempt, but no successful decolonization was achieved at the third or fourth attempt. One patient went from the uncomplicated cases protocol to the complicated cases protocol after two failures with mup/CHX treatment.

Overall, 104 out of 268 patients (39 %) were successfully decolonized, whereas 164 were not (Fig. 2). The success rates of mupi/CHX and PVPI in the first treatment attempt were 51 and 18 %, respectively, and 29 and 16 % in the second attempt, respectively.

**Fig. 3:**
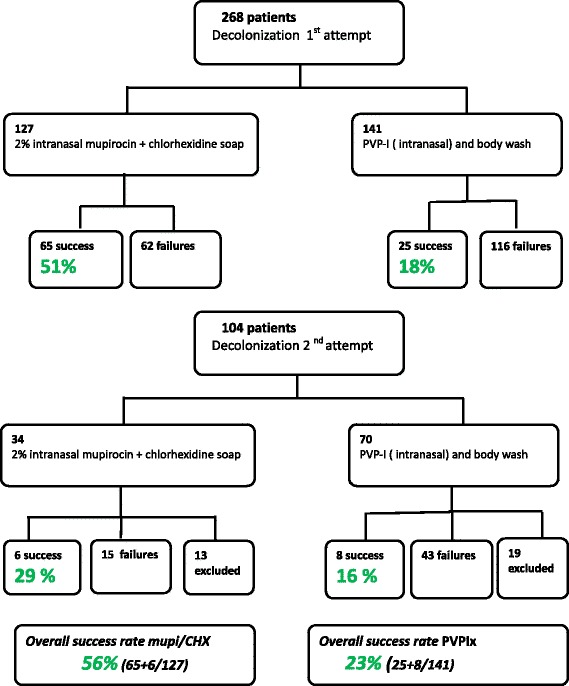
Decolonization results among the two protocols

The success rates of mupi/CHX and PVPI were, respectively, 56 and 23 % (Fig. 3).

Among the 104 patients who were successfully decolonized, 53 were re-admitted after a median period of 162 days, with 50 out of the 53 (94 %) still testing negative for MRSA carriage at the time of re-admission.

Outside our cohort, among the 328 patients who were prescribed decolonization with incomplete follow-up, 109 had at least one (85) or two (24) negative screenings for MRSA, with 185 out of these 328 patients being subsequently re-admitted to the hospital; of these individuals, 111 were negative for MRSA at the time of re-admission, while 65 were positive and 9 were not screened.

If we consider these final 111/185 patients as decontaminated with success (even if not at the time of first discharge) and add them to the 104/268 successfully treated at discharge, the rate of successful decolonization is approximately 47 % (215/453 patients).

Subsequent MRSA infection rate among the successes and failures were 0.0 and 4.8 % (8/164), respectively [*P* = 0.04]. The median (range) time period (days) from the end of treatment to infection were 43.50 (range: 2–258). Five out of 8 infected patients had been treated with the PVPI treatment and three out of 8 infected patient with the MUPI/CHX treatment.

MRSA infection rate among patients for whom decolonization treatment was unsuccessful and among patients initially excluded from decolonization treatment were 4.3 and 20 %, respectively [*P* < 0.0001]).

## Discussion

Eradication of MRSA carriage is a crucial clinical challenge, as it was demonstrated to reduce the risk of infection in MRSA-colonized patients and prevent MRSA cross-transmission to patients who were non-colonized. The efficacy of MRSA decolonization treatment remains controversial, however [10, 11]. The success rate reported in prospective trials ranged from as low as 25 % to as high as 95 % depending on the treatment used and the inclusion criteria [12–15]. Our results show that among an unselected population of patients without respiratory MRSA carriage, only one-third were successfully decolonized after the first treatment, while 39 % were decolonized following two attempts, with no further success obtained beyond two previous failures. With one exception [12], the overall success rate of MRSA decolonization in our study seems to be lower than those of other published studies [6, 16–21], one study has approximatively the same success rate and included outpatients with skin problems [22]. The success rates of mupi/CHX and PVPI in the first treatment attempt were 51 and 18 %, respectively, 29 and 16 % in the second attempt, respectively. The success rates of mupi/CHX and PVPI were, respectively, 56 and 23 % but PVPI use was restricted to complicated cases (Fig. 3).

However, by comparison with others, we included more patients with less-than-ideal prognostic factors, such as those with chronic wounds. Indeed, one-third of our patients had skin lesions, which are considered to be a contraindication for eradication measures in several studies [14, 19] and may have contributed to the comparatively low success rate. When considering only patients with carriage limited to nasal cavities, efficacy of decolonization appears significantly higher (73 %) and more in line with the range found in other studies in the literature. In addition, because of the relatively short length of hospital stay, we were unable to evaluate treatment success in many patients, which further contributes to our lower success rate. Our data highlight the gap that exists between controlled studies and real-life situations with many factors contributing to this difference.

Beyond the poor prognosis factors and the underestimation due to patients discharged from the hospital before completion of the decolonization protocol or follow-up screening, our data were also negatively impacted by our decision to reject patients for whom the hospital stay was shorter than the five days needed for decolonization. We most likely could treat a much higher number of patients by treating all concerned carriers regardless of their scheduled length of stay. The efficacy of the decolonization protocol would also be enhanced through the inclusion of oral mouth CHX solution and hair shampoo treatments and by measuring compliance to the prescribed schedule of the nurses charged with administering the protocol.

Although we observed a much lower efficacy of treatment with PVPI than with mupi/CHX in eradicating MRSA occurrence, patients who received the two treatments differed considerably in terms of their risk of failure. Thus, the question of what constitutes the best choice of topical MRSA decolonization treatment remains unresolved. We continue to avoid the use of mupirocin in patients with high levels of colonization because of the well-documented occurrence of resistance associated with the frequent use of mupirocin.

The rate of MRSA infection was found to be lower even in patients who were treated without success than in untreated patients. We should also highlight that patients excluded from decolonization (174 patients) have a high infection risk. Attempted decolonization would thus seem to be at least a somewhat effective approach for reducing MRSA infection even in patients without effective eradication. Because of this, we recommend that systematic topical decolonization in MRSA carriers be undertaken, in accordance with recent proposals made by other researchers [13].

Five factors were associated with a high risk of failure of the decolonization treatment: presence of wounds, presence of more than 2 colonized sites, other situations than only being positive for MRSA by nasal swab, and where MRSA resistance to mupirocin was high (Table 2). The following clinical conditions did not affect the decolonization rate: diabetes, immunosuppressive therapy, presence of a urinary catheter, dialysis, COPD, or presence of a central venous catheter.

**Table 2:** Characteristics of decolonized patients with successful and unsuccessful decolonization outcome (*n* = 268)

	Successful decolonization	Unsuccessful decolonization	OR (95 % CI)	*P*
	(*n* = 104)	(*n* = 164)		
Presence of wound	14 (13.64)	71 (83.53)	4.9 (2.5–9.3)	<.00001
≥2 sites	38 (36.5)	90 (54.8)	0.4 (0.2–0.7)	.003
Only nose	30 (28.8)	11 (6.71)	0.3 (0.2–0.6)	<.00001
Mupirocin resistance	3 (2.8)	18 (10.98)	4.1 (1.1–14.4)	.001
Mupirocin R (high level of resistance)	1 (0.96)	8 (4.88)	5.2 (0.65–42.8)	<.00001
Mupirocin I (low level of resistance)	2 (1.9)	10 (6.1)	3.3 (0.7–15.4)	.10
Subsequent MRSA Infection	0 (0)	8 (4.8)	Undefined	.002

Time period: days from the end of treatment to infection average: 76.25 median: 43.50 (rang: 2–258)

*Note*: data are no (%) of patients

## Conclusion

Our results fit well with the prescription of decolonization based on local strategy protocols but reflect a low rate of successful treatment compared to that of other studies in the literature. This may reflect differences in prevalence of failure risk factors compared with our population. To improve the results of decolonization, we suggest to ensure compliance of the nursing and medical staff to the care protocol (not assessed in our study) and that an oral decolonization component should be added to the decolonization procedures. In addition, the MRSA infection rate differed significantly between the group of patients successfully decolonized and those who were not and even between the instance of unsuccessful decolonization and patients who were not infected with MRSA.

Finally, although the success rate of decolonization was not high in our study, the effectiveness of decolonization on the infection rate, justifies the continuation of this strategy, even if a marginal cost is incurred.

## References

[1] Klevens RM, Morrison MA, Nadle J, Petit S, Gershman K, Ray S (2007). Invasive methicillin-resistant Staphylococcus aureus infections in the United States. JAMA.

[2] Gemmel CG, Edwards DI, Fraise AP, Gould FK, Ridgway GL, Warren RE (2006). Guidelines for prophylaxis and treatment of methicillin-resistant Staphylococcus aureus (MRSA) infections in the UK. J Antimicrob Chemother.

[3] Kluytmans J, van Belkum A, Verbrugh H (1997). Nasal carriage of *Staphylococcus aureus*: epidemiology, underlying mechanisms, and associated risks. Clin Microbiol Rev.

[4] Huang S, Platt R (2003). Risk of methicillin-resistant Staphylococcus aureus infection after previous infection or colonization. Clin Infect Dis.

[5] Kalmeijer MD, van Nieuwland-Bollen E, Bogaers-Hofman D, de Baere GAJ, Kluytmans JAJW (2000). Nasal carriage of Staphylococcus aureus is a major risk factor for surgical-site infections in orthopedic surgery. Infect Control Hosp Epidemiol.

[6] Ridenour G, Lampen R, Federspiel J, Kritchevsky S, Wong E, Climo M (2007). Selective use of intranasal mupirocin and chlorhexidine bathing and the incidence of methicillin-resistant *Staphylococcus aureus* colonization and infection among intensive care unit patients. Infect Control Hosp Epidemiol.

[7] Robicsek A, Beaumont JL, Thomson RB, Govindarajan G, Peterson LR (2009). Topical therapy for methicillin-resistant *Staphylococcus aureus* colonization: impact on infection risk. Infect Control Hosp Epidemiol.

[8] Van Rijen M, Bonten M, Wenzel R, Kluytmans J (2008). Mupirocin ointment for preventing Staphylococcus aureus infections in nasal carriers. Cochrane Database Syst Rev..

[9] Harbarth S, Dharan S, Liassine N, Herrault P, Auckenthaler R, Pittet D (1999). Randomized, placebo-controlled, double-blind trial to evaluate the efficacy of mupirocin for eradicating carriage of methicillin-resistant Staphylococcus aureus. Antimicrob Agents Chemother.

[10] Loeb M, Main C, Walker-Dilks C, Eady A. Antimicrobial drugs for treating methicillinresistant Staphylococcus aureus colonization. Cochrane Database Syst Rev. 2003(4):CD003340 81. 10.1002/14651858.CD003340 PMC12334153 14583969

[11] Loveday HP, Pellowe CM, Jones SRLJ, Pratt RJ (2006). A systematic review of the evidence for interventions for the prevention and control of methicillinresistant Staphylococcus aureus (1996-2004): report to the Joint MRSAWorking Party (Subgroup A). J Hosp Infect..

[12] Harbarth S, Liassine N, Dharan S, Herrault P, Auckenthaler R, Pittet D (2000). Risk factors for persistent carriage of methicillin-resistant Staphylococcus aureus. Clin Infect Dis.

[13] Huang SS, Septimus E, Kleinman K, Moody J, Hickok J, Avery TR (2013). Targeted versus universal decolonization to prevent ICU infection. N Engl J Med.

[14] Gilpin DF, Small S, Bakkshi S, Kearney MP, Cardwell C, Tunney MM (2010). Efficacy of a standard methicillin-resistant Staphylococcus aureus decolonisation protocol in routine clinical practice. J Hosp Infect.

[15] Kampf G (2004). The value of using chlorhexidine soap in a controlled trial to eradicate MRSA in colonized patients. J Hosp Infect.

[16] Rohr U, Mueller C, Wilhelm M, Muhr G, Gatermann S (2003). Methicillin-resistantStaphylococcus aureus whole-body decolonization among hospitalized patients with variable site colonization by using mupirocin in combination with octenidine dihydrochloride. J Hosp Infect.

[17] Marschall J, Mühlemann K (2006). Duration of methicillin-resistant Staphylococcus aureus carriage, according to risk factors for acquisition. Infect Control Hosp Epidemiol.

[18] Buehlmann M, Frei R, Fenner L, Dangel M, Fluckiger U, Widmer AF (2008). Highly effective regimen for decolonization of methicillin-resistant Staphylococcus aureus carriers. Infect Control Hosp Epidemiol.

[19] Kohler P, Bregenzer-Witteck A, Rettenmund G, Otterbech S, Schlegel M (2013). MRSA decolonization: success rate, risk factors for failure and optimal duration of follow-up. Infection.

[20] Dow G, Field D, Mancuso M, Allard J. Decolonization of methicillin-resistantStaphylococcus aureus during routine hospital care: Efficacy and long-term follow-up. Can J Infect Dis Med Microbiol. 2010 Spring;21(1):38–44. 10.1155/2010/590530 PMC2852281 21358884

[21] Ammerlaan HS, Kluytmans JA, Berkhout H, Buiting A, de Brauwer EI, van den Broek PJ (2011). Eradication of carriage with methicillin-resistant Staphylococcus aureus: effectiveness of a national guideline. J Antimicrob Chemother.

[22] Meyer V, Kerk N, Mellmann A, Friedrich A, Luger TA, Goerge T (2012). MRSA eradication in dermatologic outpatients - theory and practice. J Dtsch Dermatol Ges.

